# Diffuse purpuric eruption in a patient with acute myeloid leukemia

**DOI:** 10.1016/j.jdcr.2023.09.007

**Published:** 2023-09-24

**Authors:** Zoha K. Momin, Aya Alame, Travis Vandergriff, Cristina Thomas, Kaveh Nezafati

**Affiliations:** Department of Dermatology, University of Texas Southwestern Medical Center, Dallas, Texas

**Keywords:** acute myeloid leukemia, Ara-C, cytarabine, cytarabine syndrome, purpura, purpuric eruption, purpuric papular eruption

## Case presentation

**Fig 1:**
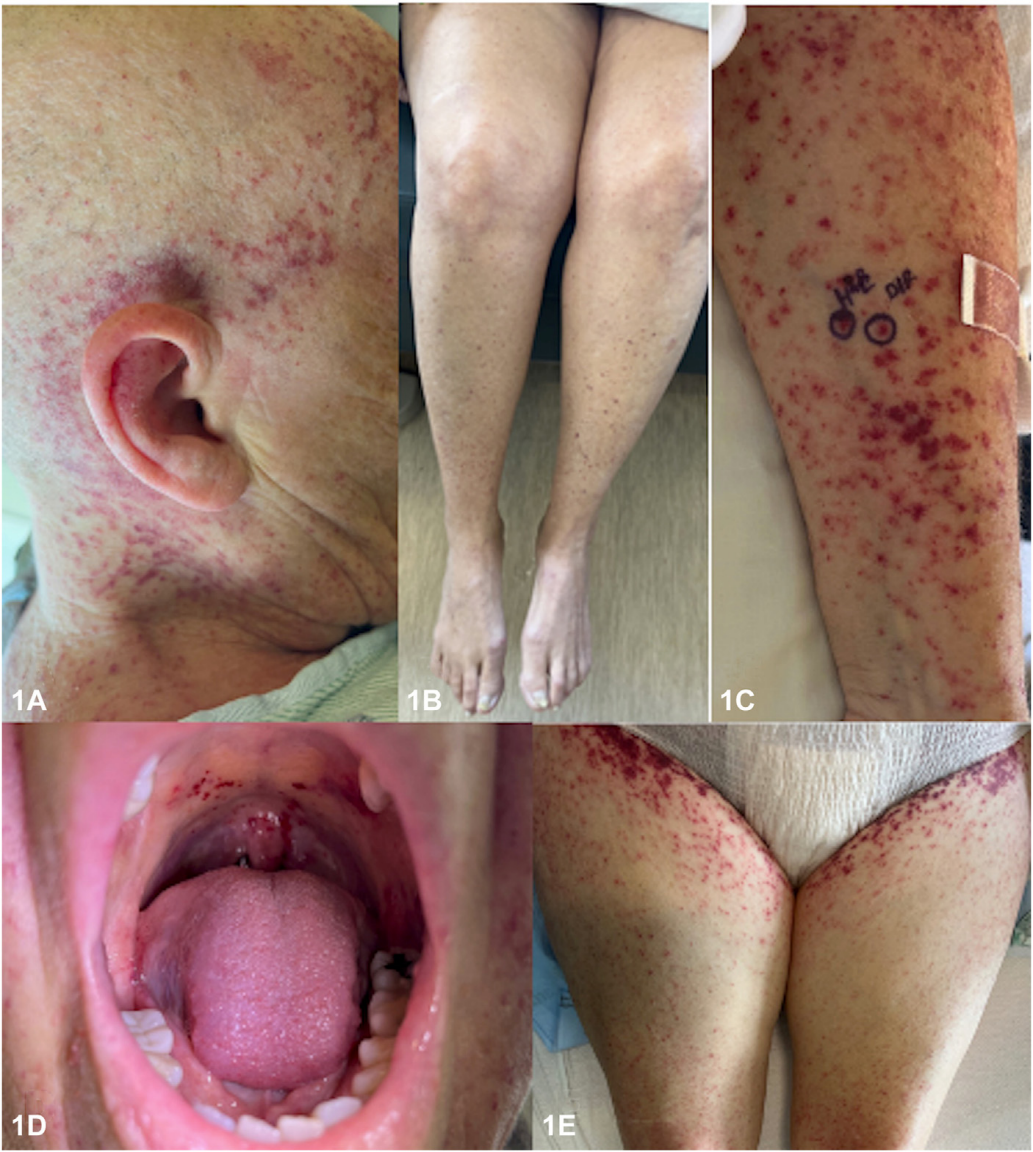

**Fig 2:**
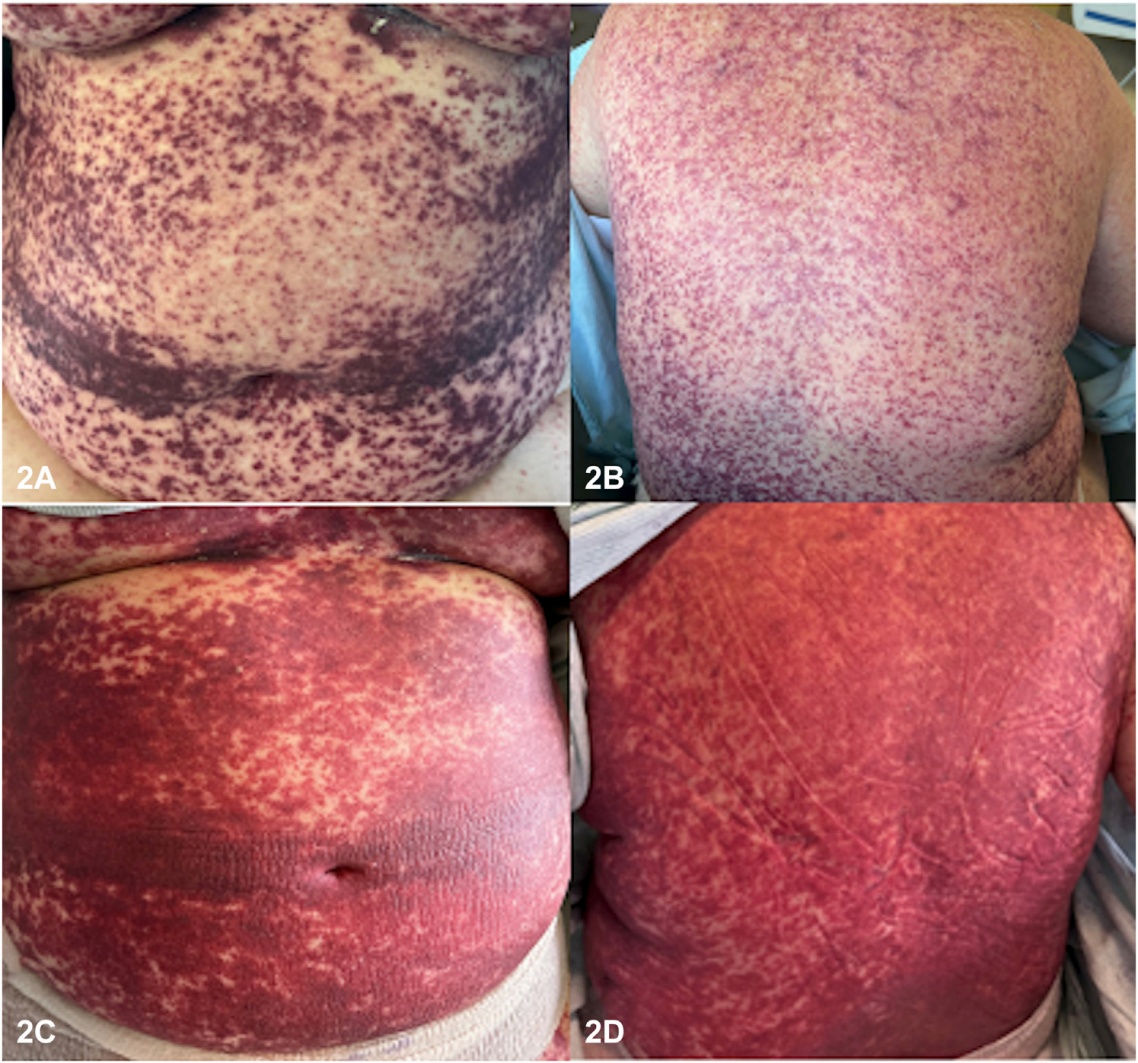

**Fig 3:**
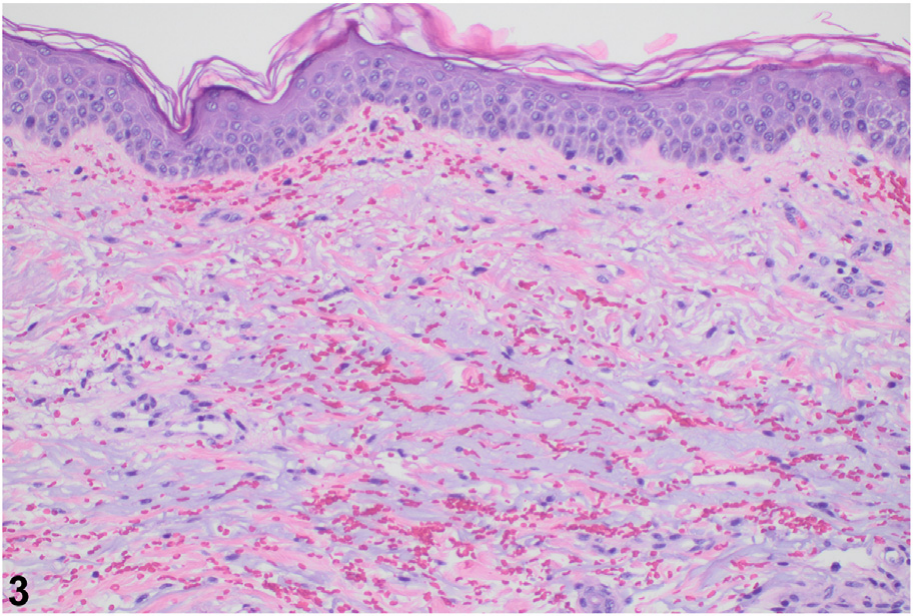

A 67-year-old Caucasian female newly diagnosed with acute myeloid leukemia (AML) was initiated on chemotherapy with daunorubicin 60 mg/m^2^ (days 1-3) and cytarabine 100 mg/m^2^ (days 1-7). The patient developed a progressive, purpuric eruption on day 20 post-treatment initiation. She had diffuse, purpuric macules involving her trunk, chest, extremities, face, scalp, and palate (Fig 1). Her eruption became more confluent in the days following her initial presentation (Fig 2). Bloodwork revealed severe pancytopenia with negative autoimmune markers, infectious work-up, and normal complements. Punch biopsy revealed extravasated red blood cells in the superficial dermis without any vasculitis or inflammation (Fig 3).

**Question 1: What is the most likely diagnosis?**

A. Cutaneous vasculitis
B. Fixed drug eruption
C. Viral exanthem
D. Progressive papular purpuric eruption
E. Neutrophilic eccrine hidradenitis

**Answers:**

A. Cutaneous vasculitis – Incorrect. While both vasculitis and progressive papular purpuric eruption associated with cytarabine may appear clinically similar, the absence of vasculitis on histology points away from this diagnosis.^1^
B. Fixed drug eruption – Incorrect. The diffused involvement of this eruption, lesion morphology, and absence of histologic evidence rule out this diagnosis.^2^
C. Viral exanthem – Incorrect. Although immunosuppressed, this patient did not have any other clinical signs or symptoms indicative of viral infection, nor was her infectious work-up remarkable. Further, histologic assessment was not consistent with this diagnosis.^2^
D. Progressive papular purpuric eruption – Correct. A frequently used antineoplastic for hematologic malignancies, cytarabine can cause a progressive papular purpuric eruption that develops during or days to weeks after treatment. Unlike other more serious dermatoses induced by cytarabine use, this purpuric eruption shows evidence of hemorrhage and purpura with several possible concomitant features such as spongiosis, acantholysis and dyskeratosis, and/or lymphocytic infiltrate on biopsy. Patients often also have evolution of their eruption with involvement of the skin folds.^2^^,^^3^
E. Neutrophilic eccrine hidradenitis – Incorrect. While most associated with cytarabine, neutrophilic eccrine hidradenitis presents with sterile neutrophilic infiltrate in the dermis.^1^^,^^3^^,^^4^

**Question 2: Which of the following risk factors is associated with increased incidence of the above described and other cytarabine-associated cutaneous reactions?**

A. Patient age >50 years of age
B. Underlying diagnosis of AML
C. Concurrent steroid use
D. Lower cytarabine doses
E. Female sex

**Answers:**

A. Patient age >50 years of age – Incorrect. While cytarabine-related papular purpuric eruptions and other toxicities have been observed across a wide range of patient ages, they occur more commonly in patients <50 years of age independent of other characteristics.^5^
B. Underlying diagnosis of AML – Correct. Compared to other hematologic malignancies including non-Hodgkin’s lymphoma, myelodysplastic syndrome, and acute lymphoblastic leukemia, patients with AML have been noted to have significantly higher rates of cutaneous reactions to cytarabine.^5^
C. Concurrent steroid use – Incorrect. Patients that receive steroids as part of their chemotherapy regimen or infusion pretreatment have significantly fewer adverse cutaneous reactions including cytarabine-related papular purpuric eruptions.^5^
D. Lower cytarabine doses – Incorrect. Although cytarabine associated cutaneous reactions have been observed in patients that are cytarabine- naïve or on low-dose therapy, cytarabine-related papular purpuric eruptions have been most often noted in patients on high dose therapy as indicated for hematologic malignancies like AML.^3^^,^^5^
E. Female sex – Incorrect. Similar rates of cutaneous toxicity to cytarabine have been observed when comparing male and female patients.^5^

**Question 3: Which of the following treatment strategies is most suitable for this diagnosis?**

A. Systemic steroids
B. Symptomatic treatment
C. Chemotherapy cessation
D. Photopheresis
E. Doxycycline

**Answers:**

A. Systemic steroids – Incorrect. Unlike cytarabine syndrome and more serious drug reactions like Stevens-Johnson syndrome, cytarabine-associated papular purpuric eruptions do not require systemic steroid therapy if symptoms are well-controlled with topicals agents alone.^2^
B. Symptomatic treatment – Correct. Cytarabine-induced papular purpuric eruptions are self-limited and resolve within 3-20 days after onset with no further sequalae. Treatment is centered around symptomatic management of pruritus and burning with topical corticosteroids, emollients, and antihistamines as needed. An additional short oral steroid taper may be prescribed in cases where patients are severely symptomatic.^2^^,^^3^
C. Chemotherapy cessation – Incorrect. While fueled by an interplay of hypersensitivity, immune-mediated effects, and direct cell toxicity, cytarabine-associated papular purpuric eruptions do not preclude patients from future cytarabine therapy.^2^ Patients can often continue or successfully rechallenge cytarabine without rash reappearance.^3^^,^^4^
D. Photopheresis – Incorrect. Photopheresis targets aberrant white blood cells which do not directly underlay the pathogenesis of cytarabine-associated papular purpuric eruptions.^2^^,^^3^
E. Doxycycline – Incorrect. With both anti-inflammatory and antimicrobial properties, doxycycline has been used to prophylactically prevent skin toxicity across a variety of cancer therapies; however, it has not been indicated for use in cutaneous reactions associated with cytarabine chemotherapy.^2^, ^3^, ^4^

## Conflicts of interest

None disclosed.
